# Supplementary material for: Transcriptome Analysis Reveals Vimentin-Induced Disruption of Cell–Cell Associations Augments Breast Cancer Cell Migration
Source: Cells. 2022 Dec 13;11(24):4035. doi: 10.3390/cells11244035 (PMC9776984; doi:10.3390/cells11244035)
Supplement: Supplementary file 1 [file cells-11-04035-s001.zip › DEGs and enrichment analysis.zip!/DEGs and enrichment analysis/WT_VIMvsVector GO enrichment/UP/WT_VIMvsVector_up.dot.pdf]

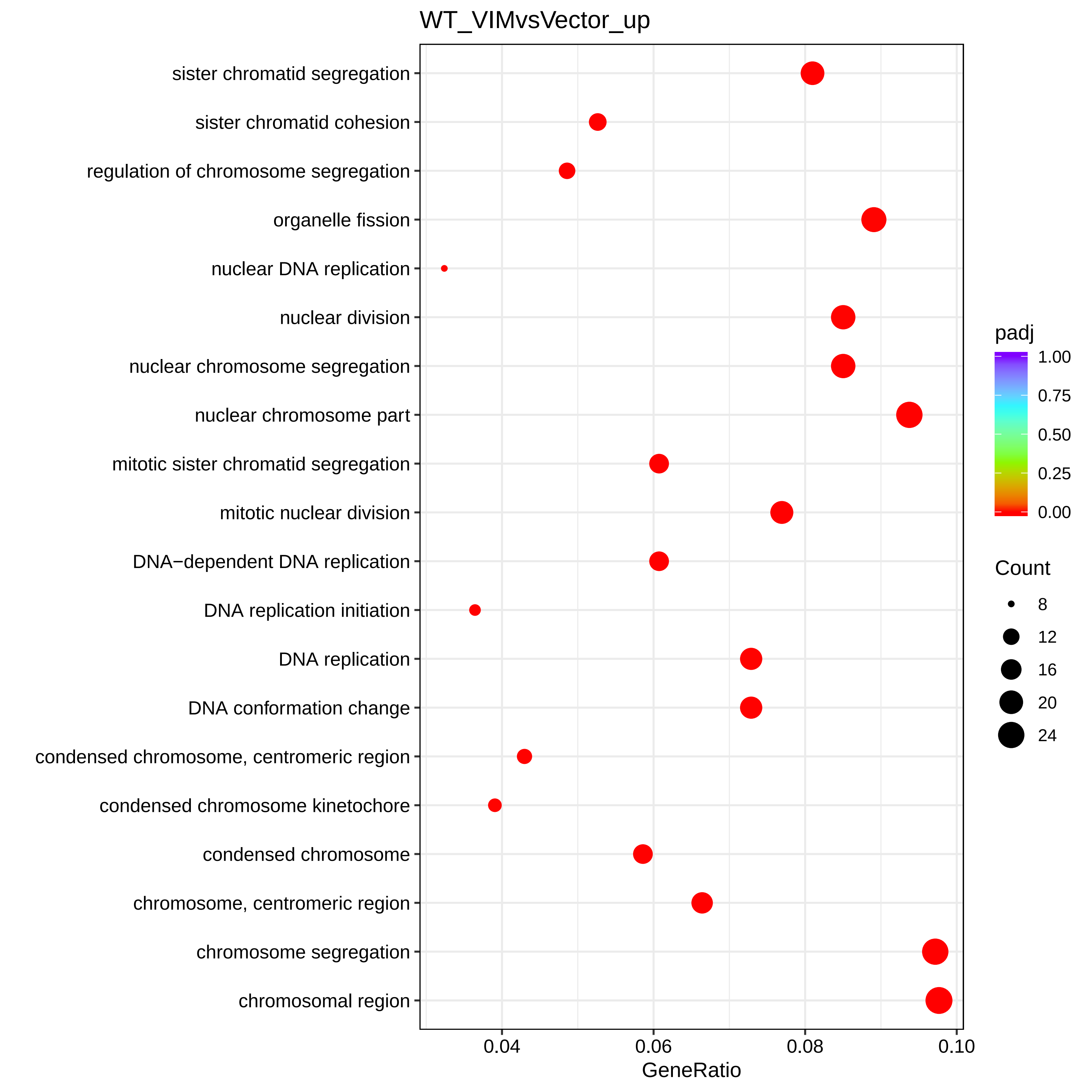

WT_VIMvsVector_up
sister chromatid segregation
sister chromatid cohesion
regulation of chromosome segregation
organelle fission
nuclear DNA replication
nuclear division
nuclear chromosome segregation
nuclear chromosome part
mitotic sister chromatid segregation
mitotic nuclear division
DNA−dependent DNA replication
DNA replication initiation
DNA replication
DNA conformation change
condensed chromosome, centromeric region
condensed chromosome kinetochore
condensed chromosome
chromosome, centromeric region
chromosome segregation
chromosomal region
0.04
0.06
0.08
0.10
GeneRatio
padj
1.00
0.75
0.50
0.25
0.00
Count
8
12
16
20
24
